# Editorial: Women in cardiovascular epidemiology and prevention

**DOI:** 10.3389/fcvm.2026.1791590

**Published:** 2026-02-16

**Authors:** Stefania Triunfo, Angela Sciacqua

**Affiliations:** 1 Department of Obstetrics and Gynecology, ASST Santi Paolo Carlo, University of Milan, Milan, Italy; 2 Department of Medical and Surgical Sciences, University Magna Graecia of Catanzaro, Catanzaro, Italy

**Keywords:** adulthood, cardiovascular epidemiology, pregnancy, prevention, women

## Introduction

Cardiovascular medicine is increasingly challenged to move beyond siloed models of disease and toward frameworks capable of capturing risk, adaptation, and resilience across the life course. Traditional approaches—largely centered on established clinical endpoints and short-term risk prediction—are no longer sufficient to explain the heterogeneous trajectories of cardiovascular health observed across sex, age, lifestyle, and social context.

The present Research Topic was conceived to address this complexity by bringing together contributions that examine cardiovascular risk from complementary yet interconnected perspectives: vascular biology, autonomic regulation, structural cardiac remodeling, lifestyle and integrative interventions, and social determinants of health. Although diverse in design and population, the six articles included in this Special Issue converge on a shared premise: cardiovascular risk is cumulative, multidimensional, and often detectable long before overt disease manifests. Of particular interest is the evaluation of critical stages across the female life course, from adolescence through pregnancy to older age. By integrating conventional and complementary approaches, this Research Topic underscores the need for earlier, more personalized, and more inclusive strategies for the prevention and management of cardiovascular disease.

## Vascular dysfunction as an early and persistent signal

Hypertensive disorders of pregnancy (HDP), and preeclampsia in particular, are now firmly recognized as sex-specific cardiovascular risk enhancers. The Perspective article by Palatnik and Kulinski synthesizes compelling evidence that vascular dysfunction is not merely a transient pregnancy-related phenomenon but a process that may precede disease onset and persist long after delivery (Palatnik and Kulinski). By focusing on non-invasive vascular assessments—such as brachial artery flow-mediated dilation, arterial stiffness indices, carotid intima-media thickness, and angiogenic biomarkers—the authors underscore the potential of vascular phenotyping to improve early risk stratification in women with a history of HDP.

This opens an important area for reflection, allowing pregnancy to be considered a cardiovascular stress test capable of unmasking latent vascular vulnerability. This is particularly relevant in the current scientific context, where a significant gap exists between research tools and clinical implementation, underscoring the need for longitudinal studies that link vascular dysfunction during and after pregnancy to feasible preventive strategies.

## Autonomic regulation and lifestyle-based modulation

Autonomic imbalance is increasingly recognized as a unifying mechanism linking cardiovascular disease, metabolic dysfunction, and psychosocial stress. Two contributions in this Research Topic address autonomic regulation from complementary angles.

Zhang et al. present a comprehensive systematic meta-analysis evaluating the impact of long-term exercise interventions on heart rate variability (HRV), a key non-invasive marker of autonomic nervous system function. Their findings demonstrate that sustained exercise—particularly aerobic and resistance training—significantly improves autonomic balance, as reflected by reductions in the LF/HF ratio (Zhang et al.). Notably, the benefits appear more pronounced in individuals with existing health conditions and with interventions lasting at least eight weeks. This work strengthens the evidence base supporting exercise not only as a metabolic or hemodynamic intervention, but also as a modulator of neurocardiac regulation.

From a therapeutic perspective, Jun et al. explore autonomic and survival outcomes through a large nationwide cohort study examining acupuncture exposure in patients with heart failure. Using real-world data and robust propensity score matching, the authors report lower circulatory and all-cause mortality among patients receiving acupuncture, with evidence of a dose–response relationship (Jun et al.). While causality cannot be definitively established, this study opens an important dialogue on the role of integrative therapies in chronic cardiovascular disease management and underscores the value of large-scale observational data in generating clinically relevant hypotheses.

## Structural adaptation: physiological vs. pathological remodeling

Structural adaptation also plays a key role, particularly in young and athletic populations where distinguishing adaptive from maladaptive cardiac remodeling remains a key challenge in cardiovascular medicine. Kim et al. address this issue by examining echocardiographic features of elite adolescent Asian female soccer players, a group historically underrepresented in sports cardiology research. Their findings demonstrate early structural and functional adaptations consistent with an athlete's heart phenotype, including increased chamber size and enhanced diastolic function, while preserving normal systolic performance (Kim et al.).

This study contributes important normative data for a specific demographic group and emphasizes that physiological remodeling can begin in adolescence. It also highlights the necessity of sex- and ethnicity-specific reference standards to avoid misclassification of physiological adaptation as pathology.

## Social determinants and refined risk prediction

Beyond biological and functional markers, cardiovascular risk is deeply shaped by social context. Dienhart et al. provide compelling evidence that educational status—an easily obtainable and cost-free variable—is independently associated with subclinical atherosclerosis and improves the predictive performance of the SCORE2 risk model. By demonstrating that lower educational status is linked to higher odds of carotid plaque presence, even after adjustment for traditional risk factors, this study reinforces the importance of integrating socioeconomic determinants into cardiovascular risk assessment (Dienhart et al.).

This contribution serves as a reminder that precision cardiovascular medicine cannot rely solely on biomarkers and imaging, but must also account for the structural inequalities that influence exposure, behavior, and access to care.

### Toward an integrated cardiovascular risk framework

Taken together, the articles in this Research Topic illustrate the multifactorial nature of cardiovascular risk and adaptation. Vascular dysfunction, autonomic imbalance, structural remodeling, lifestyle interventions, complementary therapies, and socioeconomic factors should not be viewed as isolated domains, but rather as interdependent components of a complex cardiovascular system.

The needs for earlier, more personalized and more inclusive risk assessment strategies emerge from this special issue. Pregnancy-related vascular changes, heart rate variability as a dynamic biomarker of autonomic health, physiological remodeling in young athletes, integrative approaches in chronic heart failure, and educational status as a modifier of risk prediction all point toward the same conclusion: cardiovascular prevention must extend beyond traditional boundaries.

We hope that this Research Topic will stimulate interdisciplinary dialogue and longitudinal research, encouraging clinicians and investigators to adopt integrated frameworks that better reflect the biological, behavioral, and social determinants of cardiovascular health across the lifespan.

